# 

**Published:** 1976

**Authors:** 


					Bristol Medico-Chirurgical Journal Vol. 91 (i/ii)
Contents
Human Values in Medicine
Metropolitan Anthony of Sourozh
Plasma Cell Sarcoma Complicating a Case of
Multiple Myeloma
Saud A. Sejeny and C. M. Asplin
University of Bristol Examination Results  11
Printed by F. Bailey & Son Ltd., Durs'ley, Glos.

				

